# Abundant positively-charged proteins underlie JCVI-Syn3A’s expanded nucleoid and ribosome distribution

**DOI:** 10.1371/journal.pcbi.1013898

**Published:** 2026-01-27

**Authors:** Gesse Roure, Vishal S. Sivasankar, Roseanna N. Zia

**Affiliations:** Department of Mechanical and Aerospace Engineering, University of Missouri, Columbia, Missouri, United States of America; Korea Institute for Advanced Study, KOREA, REPUBLIC OF

## Abstract

Nucleoid compaction in bacteria is commonly attributed to cytoplasmic crowding, DNA supercoiling, and nucleoid-associated proteins (NAPs). In most bacterial species, including *E. coli*, these effects condense the chromosome into a subcellular region and largely exclude ribosomes to the surrounding cytoplasm. In contrast, many *Mycoplasma*—including the *Mycoplasma*-derived synthetic cell JCVI-Syn3A—exhibit a cell-spanning nucleoid with ribosomes distributed throughout. Because *Mycoplasma* are evolutionarily distant from model bacteria like *E. coli* and have undergone extensive genome reduction, Syn3A is a natural testbed for genotype-to-‘physiotype’-to-phenotype, in which genome-encoded composition reshapes cell-scale organization. Here we show that this organization can arise from Syn3A’s unusually high abundance of positively charged proteins. We develop a coarse-grained model that explicitly and physically represents a sequence-accurate chromosome together with ribosomes and cytoplasmic proteins at physiological size, charge, and abundance. With DNA and ribosomes alone, the cell-spanning nucleoid relaxes toward a compacted state that sterically excludes ribosomes, indicating missing physics beyond polymer mechanics and excluded volume. When we include electrostatic interactions by assigning effective charges to each biomolecule, positively charged proteins dynamically enrich around ribosomes and DNA, partially screening ribosome–DNA repulsion. This charge shielding enables ribosomes to penetrate the nucleoid mesh and stabilizes a cell-spanning nucleoid consistent with experiment. This behavior is robust across parameter sweeps: DNA stiffness, heterogeneous mesh size, and crowding favor compaction, whereas electrostatics and size polydispersity promote expansion, with consequences for migration pathways within the nucleoid and thus transcription–translation dynamics. The framework is parameterized directly from genomic and proteomic composition and is transferable to other bacteria.

## Author summary

The chemical elements of DNA—its genes—carry the instructions for cells to grow and divide, but DNA is also a physical object packed into a crowded milieu, with organization that shapes how genetic information is accessed and used. In many bacteria such as *Escherichia coli*, the nucleoid is compacted, expelling ribosomes to the cell periphery, tending to separate transcription from translation. Yet in many *Mycoplasma*—including minimal cell JCVI-Syn3A—the nucleoid spans the entire cell, with ribosomes distributed throughout. How and why some genome-reduced bacteria adopt this expanded-nucleoid “physiotype” has remained unclear. To explore this, we built a whole-cell coarse-grained model that explicitly represents Syn3A DNA, ribosomes, and cytoplasmic proteins. The model confirms that DNA stiffness, DNA-binding proteins, and crowding all favor compaction. In contrast, we find that Syn3A’s unusually high abundance of positively charged proteins is a crucial ingredient for chromosome expansion. These proteins enrich around ribosomes and DNA, partially charge-shielding ribosomes, which enables them to enter the nucleoid mesh and stabilize a cell-spanning nucleoid. These results support a genotype-to-physiotype-to-phenotype link in which genome-encoded proteome composition reshapes the physical interactions that organize the cell interior.

## Introduction

The chromosomal DNA in most bacteria is a long, closed-loop molecule that packs densely within the cell but, unlike eukaryotes, is not enclosed by a nuclear membrane. Instead, it forms a mesh-like structure that defines its own well-demarcated region. This region, called the nucleoid, contains most of the chromosome and hosts many components of gene expression within the solvent-filled “pores” of the DNA mesh. Bacterial chromosome organization spans multiple length scales, from localized bends over a few base pairs, to supercoils, to macrodomains. Structure at each of these scales shapes interactions with transcription factors and other macromolecules and thereby helps regulate gene expression. At the largest scale, the nucleoid has an overall (“global”) size and envelope. In most bacteria, including *Escherichia coli* this envelope occupies only part of the cell, leaving a surrounding DNA-poor cytoplasmic region. In such cells, ribosomes are largely excluded from the nucleoid (aside from subunits) [1–4]. This exclusion tends to separate transcription within the nucleoid from translation in the surrounding cytoplasm [5], while still permitting contexts in which ribosomes localize within or near the nucleoid periphery [6], including co-transcriptional translation.

Nucleoid compactness has direct consequences for cellular process rates. Macromolecular segregation and spatial organization can generate heterogeneous transport and encounter rates, thereby modulating reaction rates [5,7,8]; one outcome is sequestration that can accelerate mRNA translation [9–11]. Nucleoid volume and packing density also vary with growth conditions [2], and gene expression can be regulated through changes in DNA structure across scales—from local bends to supercoils to global compaction—which can hinder or enhance access to specific genes. We refer to these links between physical organization and downstream biological outcomes as “physiotype-to-phenotype” connections. Prior experiments and models indicate that nucleoid compaction and expansion are driven in part by cytoplasmic crowding [12–21] and by nucleoid-associated proteins (NAPs) [22], a class of DNA-binding proteins functionally analogous to eukaryotic histones. NAPs remodel DNA mechanically by introducing bends [23], altering stiffness [23], and/or bridging distant segments [24,25]. Some of these local effects—such as bridging by H-NS—are known to influence global nucleoid compaction, as shown experimentally [26] and in simulations [27]. Despite this progress, key questions remain unresolved, including why some bacterial nucleoids span the entire cell.

In contrast to most bacteria, the nucleoid in some (though not all [28]) *Mycoplasma* spans the entire cell [29–31], with ribosomes distributed throughout—including within the nucleoid [32–35]. For the *Mycoplasma*-derived synthetic cell JCVI-Syn3A in particular, cryo-EM reconstructions report an approximately uniform ribosome distribution [34], but direct measurements of the nucleoid configuration remain limited, so nucleoid structure is often inferred from ribosome organization in modeling studies. *Mycoplasma* also encode far fewer nucleoid-associated proteins (NAPs) [33,36]; given the established role of NAPs in compacting and structuring bacterial chromosomes, this paucity has been proposed as a mechanistic explanation for the expanded-nucleoid organization. However, nucleoid structure is shaped not only by NAPs but also by the surrounding cytoplasm: crowding, depletion interactions, and electrostatics are all known to influence DNA compaction [37]. Disentangling how these factors combine to restructure DNA *in vivo* is important because nucleoid architecture can modulate the spatial coupling of transcription and translation during growth and environmental adaptation.

Computational modeling is a valuable complement to experiment, in part because it is challenging to dynamically image nucleoid restructuring *in vivo*. In this work, we develop a new computational model of a representative *Mycoplasma* cell to probe the physico-chemical interplay between DNA and cytoplasm that can produce an expanded nucleoid. Existing models of nucleoid formation and remodeling span a range of resolutions, from thermodynamic theories, to polymer bead–spring models embedded in mean-field backgrounds, to simulations in which DNA interacts with an explicitly represented cytoplasm. The primary distinction among these approaches is how directly they resolve DNA–DNA and DNA–cytoplasm interactions. We briefly review these frameworks, each of which has contributed to current understanding of nucleoid structure.

Thermodynamics-based approaches typically rationalize DNA restructuring through two related lenses: polymer collapse and phase separation. De Gennes’ foundational work on polymer collapse emphasized the role of solvent quality in driving condensation [38], motivating many subsequent theories. Post and Zimm developed an early thermodynamic description of nucleoid formation by treating distinct coiling motifs as phases [39]. De Vries extended solvent-quality ideas to include the effects of cytoplasmic crowders [40]. Odijk later proposed a liquid-like phase-separation picture in which supercoils condense via depletion interactions induced by crowders [41], consistent with experimental observations of crowder-induced compaction [12]. Because these theories are computationally inexpensive, they remain widely used and often serve as benchmarks for higher-resolution simulations [42–44]. Their key limitation is that they effectively average over local spatial structure, obscuring *local* crowding, heterogeneous accessibility, and explicit DNA interactions that can modulate gene activity.

Dynamical models of DNA span a wide range of physical resolutions, from atomistic simulations that resolve local structure to continuum theories that capture system-level, far-from-equilibrium behavior. At the smallest scales, near-atomistic and all-atom simulations resolve conformational fluctuations of DNA and associated biomolecules [45–48]. These approaches can directly interrogate mechanisms such as NAP–DNA binding that induces local bending and bridging, as well as the condensing action of ions, amino acids, and polypeptides [49–52]. Coupled with recent AI advances such as AlphaFold [53], atomistic modeling has transformed structural biology, enabling high-fidelity views of protein conformational dynamics [54–56] and of how binding events remodel local DNA structure [57]. However, atomistic simulations remain prohibitively expensive for cell-scale chromosome organization: even with coarse-graining steps, studies of DNA–protein binding typically focus on segments shorter than ∼100 bp [58], and predicting cooperative deformation of the full bacterial nucleoid is beyond the reach of all-atom models alone. Multiscale strategies that combine atomistic detail with coarse-grained representations are therefore essential for extending these insights to longer DNA and more complex assemblies [59–61].

At the opposite extreme, continuum theories describe global nucleoid behavior away from equilibrium by averaging over microscopic detail. Examples include field-theoretical descriptions of diffusion [62] and active-fluid models of chromatin transport [63]. These approaches have yielded important insights into emergent phenomena, including swelling driven by competition between coiling and diffusion in bacterial chromosomes [62] and flow-driven coalescence of chromatin in eukaryotic nuclei [63,64]. Because they do not resolve local structure, however, continuum models are not designed to connect specific molecular binding events to heterogeneous changes in nucleoid microstructure. Bridging these local and global descriptions motivates intermediate-resolution, mesoscale modeling.

Mesoscale biophysical models are well suited to provide this bridge. Coarse-grained polymer frameworks represent DNA and other macromolecules as bead–spring chains of colloid-like particles, retaining explicit excluded-volume and other local interactions while remaining tractable at chromosome length scales [65]. When embedded in an explicitly represented cytoplasm, mesoscale models can link multi-scale DNA remodeling to cell-wide biomolecular organization and transport.

Bead–spring polymer models have been used to capture a broad range of chromosome-level phenomena, including supercoiling dynamics [66–68], knotting [69], replication [70], condensation [71], and macromolecular transport within phase-separated domains [72]. This combination of local physical resolution with cell-scale reach has made mesoscale models a powerful tool for studying nucleoid compaction and cytoplasmic organization. For example, Sottas et al. [67] showed that dynamical supercoiling and salt concentration influence plasmid compaction, and Joyeux demonstrated that confinement and crowding can compact a generic bead–spring nucleoid even in the absence of supercoiling [73]. Subsequent model improvements incorporated supercoiling and DNA-bridging proteins, which were shown to induce further compaction [27,74]. Related simulations introduced additional effects such as crowder-size bidispersity and non-spherical confinement [3,4,20]. In contrast to thermodynamic theories, these approaches retain explicit local interactions with crowders and bridging proteins. A recent combined experimental and mesoscale simulation study in *E. coli* further demonstrated how nucleoid structure and charge influence macromolecular diffusion and segregation [8]. Overall, physically resolved coarse-grained models have clarified how nucleoid structure responds to cytoplasmic interactions and, conversely, how nucleoid features shape biomolecular organization.

Despite this progress, it remains unclear why the nucleoid is persistently compact in many bacteria, yet expands to fill the entire cell in others. These distinct global states correlate with markedly different cell-wide biomolecular organization, but the direction of causality is unresolved: does nucleoid architecture impose a particular spatial organization of the cytoplasm, or do cytoplasmic composition and interactions set nucleoid architecture? Two additional ingredients are especially likely to influence both compaction and segregation: electrostatic charge and size polydispersity, which together can promote clustering, condensate formation, and selective partitioning [75,76].

Recent physically resolved whole-cell models of *Mycoplasma*-derived synthetic cells achieved remarkable chromosomal detail, including 10 bp-resolution reconstructions of complete genomes and replication dynamics [70], and extensions incorporating RNA polymerase (RNAP) to visualize cell organization [77]. However, both studies model only DNA, ribosomes, and a simplified cytosol: they omit the cytoplasmic proteome, neglect electrostatic interactions, and do not include HU proteins. In addition, ribosomes are placed directly from experimental distributions; the chromosome is then assembled into the remaining space (and, in [77], RNAPs are added prior to DNA assembly). Under this construction protocol, a cell-spanning nucleoid follows unavoidably because the interior volume is effectively reserved for DNA during assembly. By contrast, in a physiological cytoplasm, the missing components—a dense, size-polydisperse proteome with heterogeneous charge—can be expected to strongly reshape both nucleoid microstructure and global extent. Indeed, Syn3A is striking in this regard: its proteome is strongly charge-skewed, with a substantially larger fraction of positively charged proteins than in *E. coli* (nearly the inverse composition), suggesting that electrostatics may be a key, genome-encoded determinant of the expanded-nucleoid physiotype. These considerations motivate incorporating both cytoplasmic proteins and electrostatic interactions into whole-cell models to directly test how composition and physical interactions jointly set nucleoid organization.

In this study, we use a mesoscale whole-cell model to test which physical mechanisms can sustain an expanded-nucleoid physiotype in a *Mycoplasma*-type cell. We focus on the *Mycoplasma*-derived synthetic minimal cell JCVI-Syn3A, engineered to retain only the genome required for life [78,79]. Recent cryo-EM reconstructions report an approximately uniform ribosome distribution in Syn3A [34], consistent with the cell-spanning nucleoid organization observed across several *Mycoplasma* [32,33,35], and motivating prior models that assume an expanded nucleoid [34,70,77]. Here we move beyond DNA–ribosome-only, charge-neutral descriptions by explicitly representing cytoplasmic proteins and incorporating HU-mediated DNA bending and electrostatic interactions, enabling a direct, mechanistic test of how genome-encoded composition reshapes nucleoid organization. Because the model is parameterized from genomic and proteomic composition, the framework is readily transferable to other bacteria as such data become available [80].

## Results and discussion

### Recapitulating experiment-matched models

**Fig 1:**
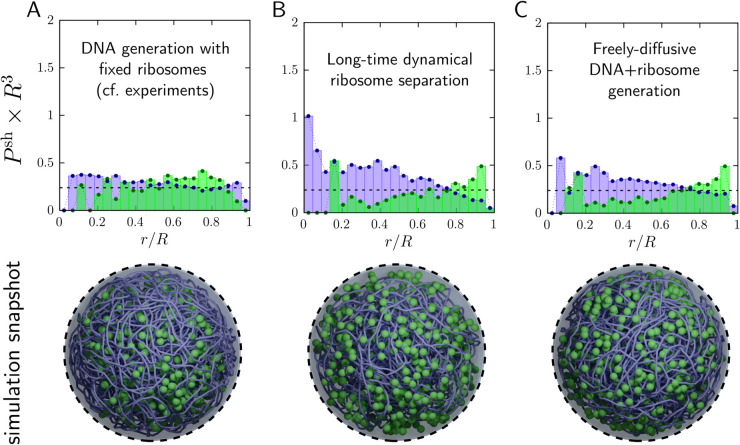
Nucleoid and ribosome spatial configurations predicted by our simplest computational model of JCVI-Syn3A, containing only DNA and ribosomes (both charge neutral). Following the ribosome data reported by [34], this model includes 503 ribosomes and a chromosome coarse-grained at 100 bp/bead. Top row: Probability per unit volume of finding a DNA bead (purple) or ribosome (green), binned into annular shells from the center (*r*/*R* = 0) to the membrane (*r*/*R* = 1) in simulations (total volume fraction ϕ=0.099). (A) Experiment-informed initial condition (no dynamics), constructed to match [34]. Both DNA and ribosomes appear approximately homogeneous throughout the cell. (B) Same initial condition as (A) after Brownian dynamics with entropic exclusion. DNA compacts toward the center and ribosomes redistribute toward the periphery, demonstrating that a DNA–ribosome-only, charge-neutral model does not sustain the experimentally inferred organization. (C) Self-assembled initial condition generated without experimental placement: DNA beads and ribosomes are initialized uniformly, after which DNA is assembled into a single closed loop via the swelling Monte Carlo algorithm (see *Methods*). The system again relaxes toward compaction and ribosome redistribution, showing that the outcome is robust to the initialization protocol and reinforcing that DNA and ribosomes alone are insufficient to recover the experimentally inferred homogeneous distribution. Bottom row: Simulation snapshots with 503 ribosomes and a sequence-accurate nucleoid, coarse-grained to 100 bp/bead (smoothed for visual clarity).

As a starting point, we asked whether the experiment-matched spatial organization reported for Syn3A can be sustained by the simplest physically resolved model containing only DNA and ribosomes. We therefore began from the ribosome distribution reported by [34], which also underlies prior Syn3A reconstructions [70,77]. In those studies, ribosomes were placed according to experiment, held fixed, and the chromosome was assembled into the remaining volume. Using the same protocol with our swelling Monte Carlo algorithm, we generated an experiment-informed initial condition with an approximately uniform ribosome distribution and a cell-spanning nucleoid (Fig 1A). As in [70], this minimal system includes only DNA, ribosomes, and entropic exclusion (see *Methods*).

Gilbert et al. [34,70] do not explicitly distinguish individual ribosomes from polysomes; their cryo-EM analysis reports the total ribosome population, irrespective of whether a ribosome is translating as a single particle or as part of a polysome. Image processing in JCVI-Syn3A indicates that approximately 25–55% of ribosomes are polysomal at a given time [34]. Thus, Fig 1 should be interpreted as a coarse-grained, population-level view of the combined single-ribosome and polysomal signal relative to the nucleoid. In the present work, we model ribosomes as independent coarse-grained macromolecules representing this total population. This approximation allows us to focus on mesoscale organization of DNA, ribosomes, and protein electrostatics, while explicit polysome architecture is deferred to future work. The potential contribution of polysomes is discussed in the Concluding Remarks.

This construction protocol reproduces the experimentally reported ribosome distribution by design, but it does not establish that the configuration is dynamically stable. Indeed, entropic exclusion alone is known to drive ribosome segregation and nucleoid compaction [73]. To test stability, we initiated Brownian dynamics from the experiment-informed configuration. The system rapidly relaxes to the state in Fig 1B: ribosomes migrate substantially (though not completely) toward the cell periphery, and the nucleoid moderately compacts, evidenced by a DNA-poor region near the membrane and an increased DNA density toward the cell center.

To check whether this outcome depends on the experiment-based initialization, we also generated DNA and ribosome configurations *ab initio*, without imposing the measured ribosome positions. Using our swelling Monte Carlo procedure to assemble the chromosome while initializing all particles uniformly, we obtain the configuration in Fig 1C. Strikingly, it is qualitatively similar to the dynamically evolved experiment-informed case in Fig 1B: ribosomes again accumulate near the membrane and the nucleoid becomes modestly compacted. Together, Fig 1 shows that, with only hard-sphere (entropic) interactions [70], ribosomes are naturally driven out of the nucleoid and the nucleoid compacts by at least ∼15% in volume. This behavior is inconsistent with the near-homogeneous ribosome distribution observed in Syn3A [34], indicating that a DNA–ribosome-only, charge-neutral model is missing key physics.

Syn3A contains many additional biomolecules with heterogeneous sizes and charges, as well as nucleoid-associated proteins. Beyond the proteome’s physiological packing fraction and charge distribution, Syn3A is also reported to have a low abundance of nucleoid-associated proteins (personal communication, John I. Glass, J. Craig Venter Inst.), which has been proposed as a contributor to its expanded-nucleoid organization. In the remainder of the Results, we therefore incorporate additional, physiologically motivated mechanisms and assess their effects on macromolecular organization: intrinsic DNA stiffness, HU-induced DNA bending, cytoplasmic crowding, and electrostatic interactions, and how each reshapes DNA–ribosome–protein distributions in Syn3A.

### Nucleoid stiffness can induce DNA compaction

Base-pair stacking, mediated by chemical bonding, underlies DNA’s intrinsic multiscale structure and gives rise to mesoscale material properties such as bending, stretching, and torsional stiffness [81,82]. As with synthetic polymers, these intrinsic material properties are expected to influence the nucleoid’s global size and compactness. More generally, the chromosome comprises both DNA and bound proteins that can further modify local curvature and effective stiffness. In our model, these effects are captured through the dimensionless stiffness coefficients $\sigma_{s}$ and $\sigma_{b}$, which set the chromosome’s resistance to stretching and bending, respectively (see *Methods*, Eqs (4)–(5), and Fig 8).

For the conceptual overview and experimental validation in Fig 1, we base our depiction of nucleoid and ribosome organization on the imaging [34] and modeling study of Gilbert et al. [70], which, to our knowledge, is the only work that provides spatially resolved measurements of ribosome distributions in JCVI-Syn3A together with an explicit physical model. This dataset anchors our qualitative description of chromosome–ribosome organization. In contrast, the quantitative parameters for protein identity, abundance, and net charge used in our simulations are derived from the JCVI-Syn3A proteomics of Breuer et al. [79], which provide comprehensive proteome composition but no spatial localization. Thus, Gilbert et al. supply the spatial context for Fig 1, whereas Breuer et al. supply the detailed proteomic information needed to parameterize the protein components of our model. These two datasets are therefore complementary: one constrains spatial organization, and the other constrains composition and charge. Our baseline model for the remainder of the study is thus built using data published by Breuer *et al.*, selected for its comprehensive proteomics [79] (see Table 1 in *Methods*).

**Fig 2:**
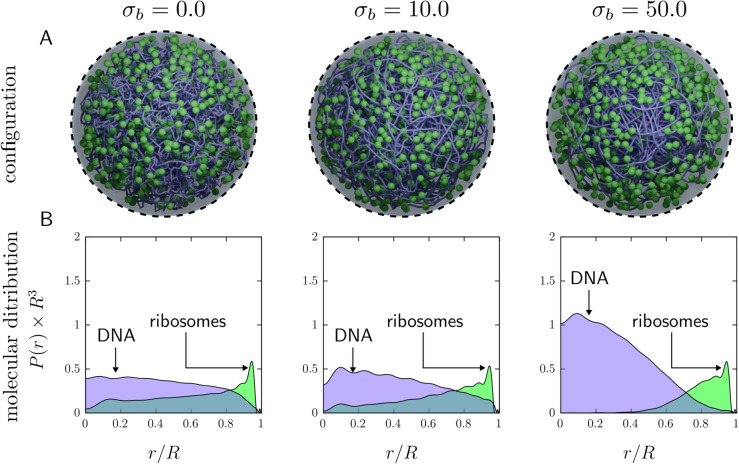
Impact of intrinsic nucleoid stiffness on the spatial distributions of DNA and ribosomes. The bending stiffness $\sigma_{b}$ (values shown at top) is applied uniformly to all DNA triplets. (A) Simulation snapshots (total volume fraction ϕ=0.079). Ribosomes: green spheres. DNA: purple (100 bp/bead; smoothed for visual clarity). (B) Radial distributions of DNA beads (purple) and ribosomes (green), from the cell center to the membrane, averaged over 100 independent simulations.

We therefore consider a baseline Syn3A model with 600 ribosomes and an intrinsic DNA bending stiffness set by base-pair stacking; explicit binding proteins are introduced in the next section. For “bare” DNA (i.e., in the absence of nucleoid-associated proteins), we set the bending stiffness coefficient to $\sigma_{b}=10$, consistent with established theoretical and experimental estimates [82]. Similar values have been used in recent coarse-grained models with simplified cytoplasmic constituents [27,83] as well as Syn3A [70]. Because changing $\sigma_{b}$ can either expand or compact the chromosome globally, we simulated a range of bending stiffness values and quantified the resulting DNA and ribosome spatial distributions. Fig 2A shows representative snapshots for a soft nucleoid ($\sigma_{b}=0$), a moderately stiff nucleoid ($\sigma_{b}=10$) corresponding to physiological conditions, and a stiff nucleoid ($\sigma_{b}=50$). To obtain adequate statistics and minimize sensitivity to knotting and kinetic trapping, we average results over 100 simulations and compute ensemble-averaged radial distributions of DNA beads and ribosomes (see *Methods*).

Fig 2B shows that intrinsic bending stiffness influences both nucleoid compactness and ribosome localization. Increasing $\sigma_{b}$ leads to a more compact chromosome and a stronger redistribution of ribosomes away from DNA-rich regions: the stiffer the nucleoid, the more ribosomes are depleted from the cell interior. Even in the softest case ($\sigma_{b}=0$), however, a DNA-poor peripheral region persists and is preferentially occupied by ribosomes. Overall, varying inherent DNA stiffness alone does not recover the near-homogeneous ribosome distribution inferred from experiment in the simplified DNA–ribosome models used here and previously [34,70]. Moreover, reducing stiffness from $\sigma_{b}=10$ to $\sigma_{b}=0$ produces only modest changes in macromolecular organization, whereas a large increase to $\sigma_{b}=50$ yields an extended DNA-depleted layer near the membrane populated almost exclusively by ribosomes, along with near-complete ribosome depletion from the cell center. We return to the mechanistic origin of these trends in later sections, where we interpret them in terms of competition between entropic exclusion and the energetic cost of bending a stiff polymer.

These results are obtained in a deliberately simplified setting—without electrostatics, without proteomic crowding, and without nucleoid-associated proteins such as HU, which are known to remodel DNA bending stiffness *in vivo* and *in vitro* [84]. Before adding these missing components, we first examine how intrinsic stiffness couples to DNA microstructure and to the ribosome distributions observed in Fig 2.

### Nucleoid pore distribution partly explains ribosome exclusion

**Fig 3:**
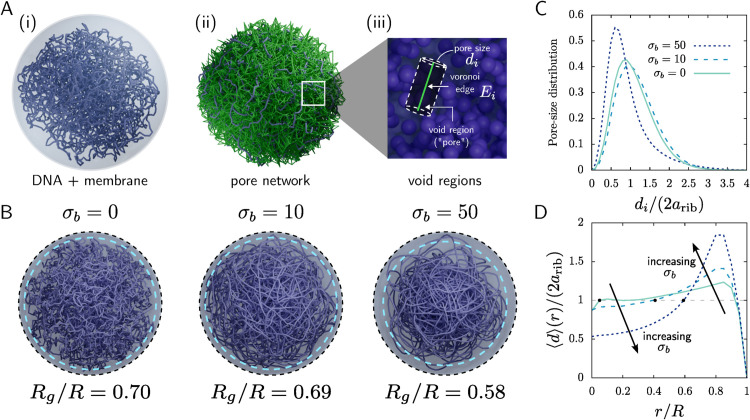
Voronoi analysis of nucleoid pores (mesh size) for three values of bending stiffness, as indicated in legends. (A) Voronoi interrogation of the nucleoid. (i) Simulation image showing only the DNA and enclosing membrane (ribosomes made invisible for visual clarity). (ii) Voronoi tessellation throughout the nucleoid. The resulting (infinitesimally thin) edges, shown in green, traverse the interconnected voids within the nucleoid. (iii) Determination of the radius *d*_*i*_ (pore size) of a void segment centered around a Voronoi edge *E*_*i*_. (B) Visualization of nucleoid structure for three values of bending stiffness. The resulting radius of gyration *R*_*g*_/*R*, normalized on total cell radius, is shown below each image. Dashed blue lines enclose 95% of DNA, with values *R*_95_/*R* = 0.93, 0.94, and 0.864 from less to more stiff. (C) Distribution of pore sizes *d*_*i*_ (normalized on ribosome diameter) in the nucleoid [85]. (D) Average size ⟨d⟩(r) of void segments located at distance *r* from the center of the cell, calculated using Eq (1). For both (C) and (D) we use data from 100 realizations.

The stiffness-dependent ribosome redistribution in Fig 2 suggests that nucleoid *microstructure*—in particular, its pore-size (mesh-size) distribution [86]—may help determine where ribosomes can reside. To quantify this porous structure, we performed a Voronoi-based analysis following [85], with modifications to account for membrane confinement. As illustrated in Fig 3A, we compute a Voronoi tessellation in the void space excluded by DNA and the confining membrane, yielding a network of edges (green) that traces connected pore pathways. Each edge *E*_*i*_ is associated with a local void segment whose characteristic diameter *d*_*i*_ sets the size of the largest spherical particle that can occupy (or traverse) that segment. We exclude edges near the membrane that produce spuriously small or ill-defined voids.

Fig 3B shows that increasing bending stiffness progressively reorganizes nucleoid microstructure, with apparent increase in persistence length, i.e. the length over which a segment extends like a rod with little curvature (see Fig B in S1 Text for persistence length results). Confinement together with stiffness produces a pronounced DNA-depleted region near the membrane, with DNA enriched toward the cell interior; this trend is amplified at larger $\sigma_{b}$, consistent with prior observations for confined semiflexible polymers [87]. Simulations of DNA alone (no ribosomes or proteins) show the same center-enriched, wall-depleted pattern (see Fig F in S1 Text), indicating that it arises from polymer mechanics under confinement rather than from excluded-volume interactions with other macromolecules. The dashed contours in Fig 3B (enclosing 95% of DNA) and the reported radius of gyration (*R*_*g*_) values further indicate that lower intrinsic stiffness expands the global nucleoid envelope.

We next quantified pore sizes across 100 realizations at each stiffness. The pore-size distributions in Fig 3C exhibit a dominant pore scale for each $\sigma_{b}$, with a weak tail toward larger pores. For the stiffest nucleoid, most pores are smaller than a ribosome diameter, consistent with the strong ribosome depletion observed at $\sigma_{b}=50$ in Fig 2. For $\sigma_{b}=0$ and $\sigma_{b}=10$, the *global* distributions are similar, despite visible differences in microstructure in Fig 3B; this similarity reflects the fact that panel (C) averages pore sizes over the entire nucleoid.

The key distinction emerges when pore sizes are resolved spatially. We therefore computed the mean local pore diameter ⟨d⟩(r) as a function of distance *r* from the cell center by sampling Voronoi edges that intersect a spherical surface *S*_*r*_ of radius *r* and averaging their associated diameters:

$$\langle d\rangle (r)=\frac{1}{N_{r}}\sum_{E_{k}\cap S_{r}\neq \emptyset }d_{k},$$ (1)

where *N*_*r*_ is the number of edges intersecting *S*_*r*_. Fig 3D shows that increasing stiffness enhances spatial heterogeneity: pores become smaller in the nucleoid interior and larger near its periphery. In particular, increasing $\sigma_{b}$ from 0 to 10 reduces ⟨d⟩(r) near the center while increasing it toward the nucleoid edge, even though the globally averaged distributions in Fig 3C change only weakly.

Together, these results suggest that ribosome exclusion is governed not simply by DNA concentration but by the stiffness-controlled mesh geometry of the nucleoid. For the stiffest nucleoid, the average pore size near the center is well below a ribosome diameter, consistent with ribosome depletion from the cell interior. For the softer nucleoids, central pore sizes are closer to ribosome scale, which may permit transient entry and trapping (see S1 Text).

### Gene accessibility, migration pathways and compactness

**Fig 4:**
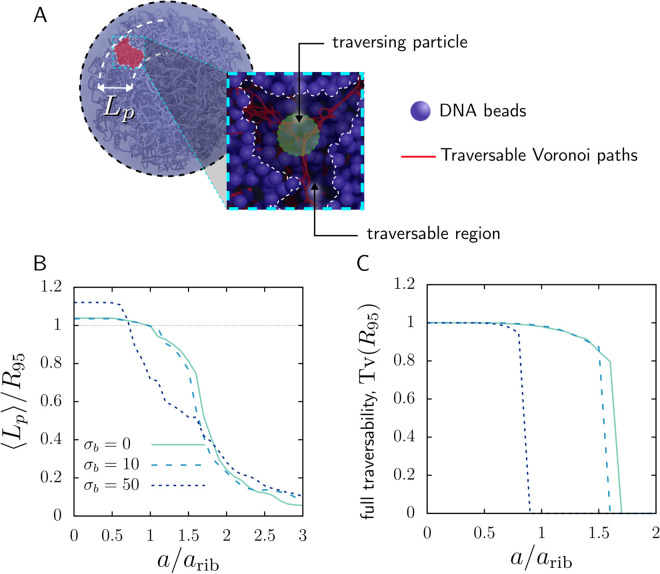
Traversability analysis of the nucleoid mesh for three values of bending stiffness $\sigma_{b}$. (A) Schematic of a biomolecule moving through a traversable region inside the nucleoid. The penetration length $\langle L_{p}\rangle$ (see annotation) quantifies the *radial* extent of that region, i.e., the fraction of the distance from center to edge of the nucleoid that is accessible along connected pore pathways. (B) Average penetration length $\langle L_{p}\rangle$ normalized by the radius enclosing 95% of DNA content, *R*_95_, as a function of macromolecular size. Angle brackets denote an average over all traversable regions (see *Methods*). (C) Fraction Tv (Eqs (19) and (20)) of pathways that are fully traversable from center to edge (i.e., $L_{p}\geq R_{95}$), giving the likelihood that a molecule can traverse the entire nucleoid.

Pore-size distributions quantify nucleoid microstructure, but they do not by themselves determine whether macromolecules can *move through* the nucleoid to reach specific loci. Long-range accessibility depends on the connectivity of pores into continuous, tortuous pathways: a molecule may fit locally yet still be unable to traverse the mesh if admissible pores do not percolate. Conversely, sufficiently small particles may explore most of the nucleoid, whereas larger particles may penetrate only partway before becoming confined to an isolated void network. To quantify this size-dependent accessibility, we use the Voronoi network constructed above and identify pathways that can be traversed by a spherical particle of radius *a*, following [85]. Specifically, we prune the Voronoi graph by retaining only edges with local diameter $d_{i}\geq 2a$ and then use a graph-traversal algorithm to extract connected components of the remaining network. Each connected component defines a *traversable region*—a portion of the nucleoid mesh within which a particle of size *a* can move without geometric obstruction (see Fig 4A). Depending on *a*, the pruned network may contain a single percolating component or multiple disconnected regions, implying either cell-scale accessibility or size-dependent trapping.

The traversable regions, penetration lengths, and full-traversability fractions reported here quantify nucleoid accessibility in a statistical ensemble sense: they characterize the connectivity of the mesh across many equilibrated configurations that a fluctuating nucleoid is expected to sample over time, rather than the lifetime of any individual pore in a single trajectory. This provides a time-averaged view of which migration pathways are typically available to molecules of a given size. Explicitly coupling this structural analysis to time-resolved chromosome–transport simulations remains an important direction for future work.

We summarize each traversable region by its penetration length *L*_*p*_, defined as the radial span accessible within that connected component (see Fig 4A). Intuitively, *L*_*p*_ measures how far a particle can move radially through connected pore space—from its most outward accessible location toward the center (and vice versa). We computed *L*_*p*_ for particle sizes spanning $0\leq a\leq 3a_{rib}$. Fig 4B shows that, on average, smaller particles penetrate deeper, and that reduced stiffness ($\sigma_{b}=0$ and 10) modestly increases penetration compared to $\sigma_{b}=50$. For all stiffness values, particles must be smaller than $\sim 1.75\;a_{rib}$ to penetrate beyond ~40% of *R*_95_, whereas very small values of $\langle L_{p}/R_{95}\rangle$ indicate effective exclusion or confinement to a small, isolated void.

Connectivity can also be summarized by the fraction of pathways that are fully traversable across the nucleoid. We therefore quantify the likelihood that a particle lies in a component with $L_{p}\geq R_{95}$, reported as Tv in Fig 4C. As in percolation phenomena [88] and in related Voronoi analyses [85], increasing particle size produces a geometric transition at a *critical size* beyond which fully traversable pathways become rare. The mild decay of Tv below unity for intermediate sizes indicates occasional trapping in disconnected pore components, whereas beyond the steep drop past the critical size, full traversal becomes unlikely. For $\sigma_{b}=0$ and 10, the critical sizes are $\sim 1.6\;a_{rib}$ and $\sim 1.75\;a_{rib}$, respectively, implying that ribosome-sized particles can often access connected pathways spanning most of the nucleoid in this simplified, charge-neutral model. In contrast, increasing stiffness to $\sigma_{b}=50$ reduces the critical size to $\sim 0.8\;a_{rib}$, consistent with strong ribosome exclusion from the nucleoid interior in Fig 2.

Overall, at the physiological stiffness modeled here for Syn3A ($\sigma_{b}=10$) and in the absence of electrostatics and cytoplasmic proteins, the nucleoid mesh remains broadly connected for a wide range of particle sizes, even though the nucleoid is globally compacted and a DNA-poor peripheral region persists.

In the next sections we incorporate additional, biologically motivated mechanisms omitted thus far—including DNA-bending proteins, cytoplasmic crowding, and electrostatics—and test how they reshape both nucleoid compactness.

### Local HU-induced bends induce further nucleoid compaction

The stiffness results above show that intrinsic DNA mechanics can compact the nucleoid and thereby bias ribosomes toward DNA-poor regions. Nucleoid architecture, however, is also shaped by DNA-binding proteins that locally remodel the polymer. Syn3A encodes only one nucleoid-associated protein (NAP), HU [89], which can stiffen DNA and induce sharp local bends [23]. Because *Mycoplasma* are reported to carry far fewer NAPs than many bacteria [36], HU scarcity has been proposed as an explanation for the expanded-nucleoid physiotype.

**Fig 5:**
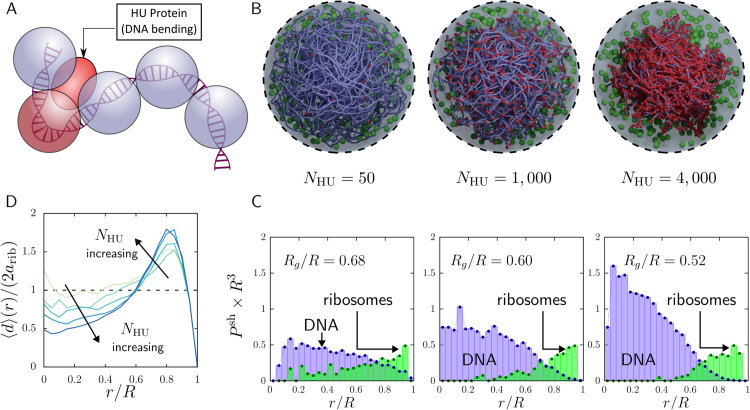
Influence of DNA-bending HU proteins on nucleoid compactness. (A) Schematic: HU binding induces a local bend characterized by a local stiffness $\sigma_{b}^{HU}$ and equilibrium angle $\theta_{HU}$ that differs from the global $\theta_{0}=\pi$. To mimic sequence-independent binding, bends are placed at random genomic locations (see *Methods*). Here we impose $\sigma_{b}=\sigma_{b}^{HU}=10$ and $\theta_{HU}=2\pi /3$ while systematically varying HU copy number $N_{HU}$ as indicated. (B) Simulation snapshots for increasing $N_{HU}$. (C) Radial distributions of DNA (purple) and ribosomes (green). (D) Mean pore diameter ⟨d⟩(r) versus radius, computed using Eq (1).

Here we test that hypothesis directly by incorporating HU-mediated bends into our model. Unlike bridging NAPs such as H-NS [27], HU primarily acts locally: depending on binding mode, it increases local stiffness and/or imposes a preferred bend angle over a short DNA segment [23] [Fig 5A]. We model HU by introducing randomly distributed local *bending defects* along the genome (see *Methods*), corresponding to a prescribed HU copy number $N_{HU}$.

Fig 5B–5D shows that increasing $N_{HU}$ progressively compacts the nucleoid, reduces pore sizes, and enhances ribosome redistribution toward the periphery—a mechanistic signature consistent with HU’s local bending action. Importantly, at the physiologically relevant HU abundance for Syn3A ($N_{HU}=50$), we observe little additional compaction relative to the bare-DNA baseline: HU at native levels does *not* further exacerbate nucleoid compaction in this simplified, charge-neutral model. Substantial compaction arises only for HU abundances well above Syn3A’s reported levels (but closer to those typical of other bacteria [36]). Thus, within our modeling assumptions, HU scarcity can be viewed as *permissive* for a cell-spanning nucleoid, but it is not, by itself, a sufficient mechanism to generate expansion.

More broadly, this HU “dose response” provides a quantitative handle for tuning nucleoid microstructure and accessibility in silico, suggesting a potential control knob for synthetic-biology design of chromosome organization. Having established that physiologically realistic HU levels do not resolve the discrepancy with experiment, we next incorporate the dominant missing component in prior Syn3A models: the remainder of the cytoplasmic proteome and its crowding effects.

### Cytoplasmic proteins entropically exclude ribosomes from the nucleoid

Up to this point, our model—as in [70]—included only DNA and ribosomes. Yet cytoplasmic proteins are a major source of crowding, and crowding is known to compact bacterial DNA through entropic (excluded-volume) effects in experiments [12,15,17,21] and simulations [73]. Here we therefore add Syn3A’s cytoplasmic proteins at physiologically relevant abundance, while still treating all macromolecules as charge neutral, to isolate the effect of protein crowding. To avoid conflating crowding with proteome-wide size polydispersity, we represent cytoplasmic proteins using a single average radius derived from the Syn3A proteome (see *Methods*).

**Fig 6:**
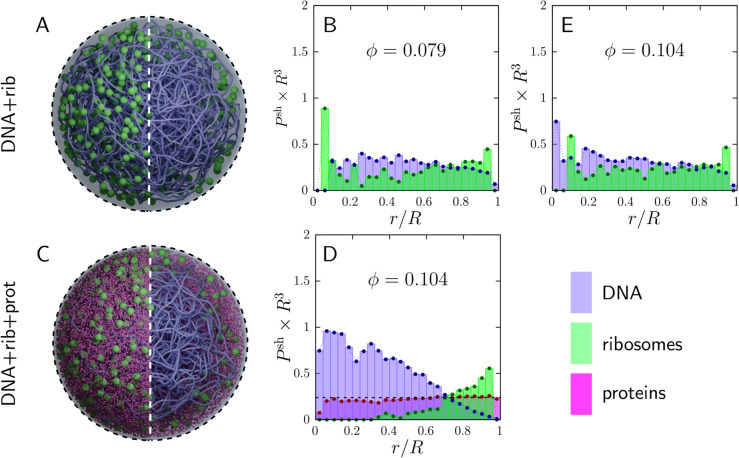
Dynamic simulations of the Syn3A model cell containing a coarse-grained chromosome and 600 ribosomes, with and without an explicit pool of cytoplasmic proteins (all macromolecules charge neutral). **Top row (DNA+rib only):** (A) & (B) show baseline model with ϕ=0.079 (middle panel of Fig 2). (A) representative simulation snapshot and (B) radial probability densities $P^{sh}(r)$ (scaled by *R*^3^) for DNA (purple) and ribosomes (green) as a function of normalized radius *r*/*R* (cell radius *R*). **Bottom row (DNA+rib+prot):** model with DNA, ribosomes, and cytoplasmic proteins at ϕ=0.104 and growth rate 0.396 db h^−1^, showing (C) a representative snapshot and (D) the corresponding profiles for DNA (purple), ribosomes (green), and proteins (pink). **Top right (E): Control simulation (DNA+rib-only)** with ϕ=0.104 for comparison to (D). The chromosome is coarse-grained at 100 bp per bead (see *Methods*); cytoplasmic proteins are modeled as monodisperse spheres with an effective size based on Breuer et al. [79].

Building on the baseline composition used above, we introduce proteins using the swelling Monte Carlo initialization (see *Methods* and S1 Text). Fig 6A and 6B recapitulates the charge-neutral DNA–ribosome model with 600 ribosomes. We then construct a more physiological composition by adding cytoplasmic proteins at the relative abundance reported for Syn3A at growth rate 0.396 db/hr [79], which increases the total macromolecular volume fraction to ϕ=10.4% (up from ϕ=7.9% in the ribosome–DNA-only baseline). The resulting configuration (Fig 6C and 6D) shows markedly stronger nucleoid compaction and a pronounced redistribution of ribosomes toward the cell periphery, accompanied by a widened DNA-depleted annulus near the membrane. Notably, the magnitude of this crowding-induced compaction is comparable to that produced by a highly stiff chromosome in Fig 2 (e.g., $\sigma_{b}=50$), underscoring that explicitly representing cytoplasmic proteins—often omitted in whole-cell simulations—substantially reshapes nucleoid organization.

A simple explanation would be that proteins compact the nucleoid merely by increasing the total volume fraction. To test whether volume fraction alone accounts for the effect, we performed a control simulation of the DNA–ribosome-only model at the same total volume fraction as the protein-containing case (Fig 6D), replacing (D)’s proteins with additional ribosomes (which are nearly five-fold larger by radius). In this control (Fig 6E), ribosomes interpenetrate the nucleoid more extensively and the nucleoid expands to accommodate them. Thus, the compaction in Fig 6C and 6D cannot be attributed solely to an increased *ϕ*; rather, it depends strongly on the presence of *smaller* crowders.

This size dependence is consistent with an entropy-driven partitioning mechanism: smaller proteins can occupy nucleoid pores, increasing local packing while maintaining substantial short-range (in-cage) motion, whereas larger ribosomes gain more long-range configurational entropy by residing in the DNA-poor periphery [90]. This points to mechanisms beyond a simple volume-fraction increase, involving a competition between entropic effects across species and the enthalpic cost of deforming the DNA polymer (stretching and bending). Here we use “vibrational (short-range) entropy” to describe local in-cage motion within pores and “configurational (long-range) entropy” to describe large-scale rearrangements and sampling of many cages, consistent with the configurational–vibrational entropy separation in statistical mechanics [91,92]. Additional simulations support this preferential partitioning and its interplay with heterogeneous mesh size (see Fig D in S1 Text): as crowding increases, proteins shift toward larger pores near the nucleoid periphery and the DNA-depleted annulus widens, with the nucleoid compacting until balanced by the DNA’s elastic energy.

Overall, adding physiologically abundant (but charge-neutral) cytoplasmic proteins drives *more* nucleoid compaction and ribosome expulsion than in the DNA–ribosome-only model. Thus, any apparent ribosome interpenetration in DNA–ribosome-only models is contingent on an incomplete cytoplasmic composition: once protein crowders are included at physiological abundance, excluded-volume interactions favor a size-dependent partitioning in which smaller proteins populate nucleoid pores while larger ribosomes redistribute toward the DNA-poor periphery. In this physiological mixture, excluded-volume interactions favor compaction rather than a stable, cell-spanning nucleoid. Because proteins, ribosomes, and DNA are charged macromolecules, we next examine how electrostatic interactions reshape these distributions.

### Effect of electrostatic interactions in nucleoid compaction and organization of cytoplasm

Notably, nearly 75% of Syn3A proteins are positively charged, with the remaining 25% negatively charged. In *E. coli*, this balance is essentially flipped: about 40% of proteins are positively charged and 60% are negatively charged. We therefore suspect that Syn3A’s unusual proteome charge distribution may influence nucleoid expansion.

To probe how electrostatics shapes molecular organization in Syn3A, we assigned each biomolecule an electrostatic surface charge (see *Methods*). For all species, we incorporated the effect of Manning counterion-condensation (Eq 7) to obtain effective charges (see *Methods*). For DNA and ribosomes, we applied uniformly distributed negative surface charge densities using experimentally determined values, either from direct measurements or inferred from structure [93,94]. Because each DNA bead represents 100 bp with radius 6nm, we accounted for coiling over this length scale by converting DNA’s linear charge density (5.9e/nm) into a bead charge: over a 12nm diameter this corresponds to −70e per 100 bp (i.e., −0.7e/bp).

For proteins, we inferred surface charge from structural data [79]. We coarse-grained the resulting patchy, pH-dependent residue-level charge patterns onto our single-bead protein representation by assigning each protein its mean surface charge (see Fig Ab in S1 Text). To isolate the qualitative effect of the proteome-wide charge distribution, we further grouped proteins by net sign and assigned group-average charges of +10e (positively charged proteins) and −7e (negatively charged proteins). The mean protein size (3nm) was taken from the same source.

Electrostatic interaction range was encoded through the Debye length, $\kappa^{-1}$, in the Debye–Hückel interaction potential (see *Methods*); we tested $\kappa^{-1}=2.2$, 1.02, and 0.68nm (discussed below). Membrane-bound proteins were not included in the model.

**Fig 7:**
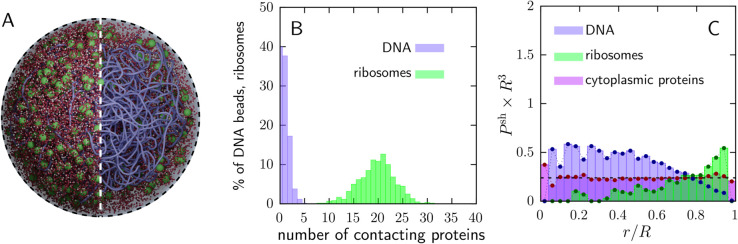
Influence of electrostatic interactions on nucleoid and cytoplasmic organization in the JCVI-Syn3A model cell. Electrostatic interactions among DNA, ribosomes, and proteins are defined in *Methods*. Results shown use Debye length $\kappa^{-1}=0.68\;nm,$ DNA charge $q_{DNA}=-70\;e$ per bead, protein charges $q_{prot,+}=+10\;e$ and $q_{prot,-}=-7\;e,$ and ribosome charge $q_{rib}=-4000\;e.$ All interactions adjusted for Manning counterion condensation as indicated in text. (A) Equilibrated whole-cell snapshot showing DNA (purple), ribosomes (green), positively charged proteins (red), and negatively charged proteins (white). (B) Fraction of DNA beads and ribosomes coated by *N* proteins (horizontal axis), averaged over all DNA beads or all ribosomes and over the time window $16a_{DNA}^{2}/D,$ where $D=kT/(6\pi \eta a_{DNA}).$ Data from a single simulation. (C) Radial distributions of DNA (purple), ribosomes (green), and proteins (pink).

Fig 7 shows the equilibrated distributions of DNA, ribosomes, and proteins in the Syn3A model cell. The snapshot in Fig 7A provides a qualitative view of molecular organization; in the right half of the image, we highlight nucleoid morphology by rendering a subset of molecules invisible. Visually, the nucleoid spans most of the cell, and (negatively charged) ribosomes are abundant deep within the (negatively charged) nucleoid. This is counterintuitive, given the ribosome’s strong negative charge, which should electrostatically repel DNA and thus bias ribosomes away from the nucleoid.

One possible explanation is suggested by the protein enrichment around both DNA beads and ribosomes, visible in the snapshot and quantified in Fig H in S1 Text. We speculate that transient protein clustering around ribosomes partially screens ribosome–DNA electrostatic repulsion, enabling ribosomes to interpenetrate the nucleoid. Syn3A’s high abundance of positively charged proteins would further promote this effect.

To quantify this putative shielding effect, we computed the number of proteins electrostatically associated with each ribosome and averaged this quantity over all ribosomes and over a window of several Brownian times, $16a_{DNA}^{2}/D,$ where $D=kT/(6\pi \eta a_{DNA}).$ We repeated the same analysis for DNA beads and report the resulting distributions in Fig 7B. The ribosome–protein enrichment should be interpreted as dynamic, electrostatically mediated recruitment rather than formation of long-lived, stoichiometric complexes.

Over this interval—long enough for a ribosome to migrate within the nucleoid—approximately 50% of ribosomes have 15--25 proteins associated with their surface. This enrichment of surrounding, positively charged proteins is expected to partially screen the ribosome’s strong negative charge. We hypothesize that such “charge shielding” explains why, in the charged model, approximately 59.3% of ribosomes dynamically reside within the nucleoid (defined here as the region enclosing 90% of the DNA) in Fig 7C, compared with 36.2% in the charge-neutral model. The resulting ribosome distribution is consistent with experimental observations in Syn3A [34] and in several *Mycoplasma* species [32,33,35].

Concomitantly, the nucleoid expands to occupy a larger fraction of the cell volume. Together, these results suggest that electrostatic shielding can drive a cell-wide ribosome distribution in Syn3A and may contribute to similar organization in other *Mycoplasmas*. They are also consistent with recent measurements in *E. coli* linking ribosome migration to coating by positively charged proteins [8,95], although the effect is expected to be weaker in *E. coli* given its lower positively charged protein-to-ribosome ratio (60:1 in *E. coli* [calculated based on data from [96]] versus 120:1 in Syn3A).

Electrostatic repulsion between ribosomes and DNA can also be reduced when positively charged proteins condense onto DNA, effectively lowering the local negative charge experienced by nearby ribosomes. We found that 8% of positively charged proteins are condensed onto DNA, defined here as residing within 10% of the combined particle radii, i.e., at separations $<1.1\;(a_{DNA}\;+\;a_{prot}).$ In contrast, negatively charged proteins show no enrichment near ribosomes beyond what is expected from Brownian diffusion.

Throughout, we use “charge shielding” to denote a reduction in the effective *short-range* electrostatic interactions between macromolecules due to nearby oppositely charged proteins. This is distinct from ionic screening, which is already encoded in the Debye–Hückel interaction via the Debye length (see *Methods*). A detailed sensitivity analysis of ionic screening and biomolecular charge, and their influence on biomolecular distributions via charge shielding, is provided in the S1 Text.

In summary, the high abundance of positively charged proteins promotes charge shielding of ribosomes, enabling them to interpenetrate the nucleoid and thereby increasing the nucleoid’s tendency to expand throughout the cell. Concomitantly, several thousand DNA beads redistribute from the cell center into an annular region near the membrane (although this shift is visually subtle in Fig 7C because the annulus occupies a substantially larger volume). The resulting organization is consistent with experimental measurements of ribosome distributions in Syn3A [34,70] and with reports of expanded nucleoids in some *Mycoplasma* species [29–31].

### Discussion and concluding remarks

Nucleoid compaction in bacterial cells is commonly attributed to DNA supercoiling, bridging by nucleoid-associated proteins (NAPs), and cytoplasmic crowding [12,22,27,41]. In many bacteria, including *Escherichia coli*, these mechanisms confine the nucleoid to a central subcellular volume. In contrast, many *Mycoplasma*—including the *Mycoplasma*-derived synthetic minimal cell JCVI-Syn3A—exhibit a cell-spanning nucleoid. This expanded-nucleoid state has been attributed to reduced levels of NAPs and other supercoiling-active proteins. Concomitantly, ribosome organization differs markedly between compacted- and expanded-nucleoid cells: ribosomes are concentrated outside the nucleoid in the former, but are distributed throughout the nucleoid (and cell) in the latter. This coupling between genome-encoded composition, physical organization, and cellular function motivates us to define ‘physiotype’ to denote a physical intermediate between genotype and phenotype. In a genotype-to-‘physiotype’-to-phenotype perspective, spatial organization shapes the opportunity for coupled transcription and translation [5], with downstream phenotypic consequences.

Recent experiments on Syn3A reported an approximately uniform ribosome distribution throughout the cell [34], motivating computational reconstructions in which ribosomes are placed according to experiment and a nucleoid self-assembles in the remaining space [70]. Syn3A is an especially well-controlled system for interrogating an expanded-nucleoid physiotype: it has a minimal genome and proteome, and its NAP complement is limited to a single type (HU). Recent discussions (personal communication, John I. Glass, J. Craig Venter Inst.) therefore raised a natural hypothesis that HU paucity is the primary driver of Syn3A’s cell-spanning nucleoid and uniform ribosome distribution. However, prior Syn3A models treated DNA and ribosomes alone and assumed charge-neutral macromolecules [70,77], motivating a more physically complete model.

We developed a Brownian dynamics model of Syn3A and reproduced the experimentally reported ribosome distribution used in prior work [34,70,77]. Using a swelling Monte Carlo algorithm [97,98], we generated an initial, self-assembled nucleoid under the same charge-neutral assumptions. Upon initiation of Brownian dynamics, however, this initialized configuration is lost: the experimentally consistent initial condition (DNA and ribosomes only, with no other proteins and no charges) is dynamically unstable, and ribosomes rapidly redistribute toward the cell periphery. This heterogeneous ribosome population is inconsistent with experiments, pointing to missing physics and motivating the refined model developed here.

We first asked whether “standard” compaction mechanisms could explain Syn3A’s cell-spanning nucleoid. Incorporating physiological DNA stiffness and HU-mediated remodeling of nucleoid microstructure (via HU binding and induced bends) consistently drove the system toward *greater* compaction, not expansion. These mechanical effects were further amplified by cytoplasmic crowding: when we added charge-neutral cytoplasmic proteins at literature-based abundance and packing fraction, the explicit excluded-volume of the proteome—absent from prior Syn3A models—*exacerbated* ribosome expulsion and nucleoid compaction once dynamics were allowed. In other words, had earlier models included the physical presence of proteins and Brownian motion, they would have robustly revealed compaction as the default outcome. The qualitative behavior reverses *only* when we include the missing ingredient: electrostatics. Endowing the proteome with a physiological distribution of electrostatic charge produces a stable, expanded nucleoid and restores a near-uniform ribosome distribution.

These results sharpen the mechanistic picture: DNA stiffness, HU-induced bending, and protein crowding all favor nucleoid compaction, whereas electrostatic interactions are the only mechanism we studied that counteracts this trend and stabilizes Syn3A’s expanded-nucleoid physiotype. The key is Syn3A’s atypical proteomic charge composition—a proteome dominated by positively charged proteins, with an unusually high ratio of positively charged proteins per ribosome (approximately doubled relative to *E. coli*). In the charged model, positively charged proteins dynamically enrich around ribosomes and DNA, and we *quantified* the resulting “charge shielding” by measuring (i) the distribution of protein association numbers around ribosomes and DNA beads and (ii) the corresponding redistribution of ribosomes and DNA across the cell volume. Consistent with this picture, ribosomes become substantially more prevalent within the nucleoid once charges are included, supporting the hypothesis that protein-mediated shielding reduces effective ribosome–DNA repulsion and allows ribosomes to interpenetrate and expand the nucleoid [8].

Our traversability analysis highlights that nucleoid mesh geometry can, in principle, constrain macromolecular transport and promote trapping within the nucleoid, even at comparable overall DNA compaction. This provides a quantitative tool for probing accessibility of genomic regions and potential links to translation organization, including proposed polysome formation deep within *E. coli*’s nucleoid [2,86]. Here we applied the analysis to an ensemble of nascent (pre-replication) configurations; extending it across growth conditions and cell-cycle stages is a natural next step.

Several extensions would help refine the mechanistic picture of electrostatically driven clustering and nucleoid organization. In the present model, protein electrostatics are intentionally coarse-grained: protein surface charge is treated as isotropic, with a single representative charge for all positively charged proteins and a second for all negatively charged proteins, and proteins are assigned a single mean size. A natural next step is to incorporate more detailed proteome heterogeneity, including distributions of protein charge and size, anisotropic (patchy) surface charge, and environmental dependence through pH and salt (Debye length), all of which can influence clustering and nucleoid organization. These refinements are particularly relevant for predictive modeling and engineering of synthetic cells. Ribosomes can also form polysomes, which can affect bacterial spatial organization [8]; incorporating explicit polysomes (rather than independent ribosomes) is therefore an important direction for future work.

The methods and frameworks developed here can be extended to incorporate additional molecular detail—including richer proteomic heterogeneity and explicit representations of mRNA translation [10,11] and transcription—and then applied to other bacterial systems.

Our framework is generalizable along two axes: the interaction physics included and the organism/cell instantiated. The pipeline that maps PDB-derived structure to coarse-grained protein size and charge can naturally be extended to include patchy surface charge and physiological size polydispersity across diverse cellular environments [11]. Likewise, the full-cell model can be parameterized from proteomics data for any bacterium where such data exist, and our genome-construction approach scales to long chromosomes (e.g., *E. coli* at 10 bp resolution). More detailed, sequence-specific biochemistry—including protein–DNA binding, supercoiling, and dynamic winding/unwinding—can be layered onto the same mesoscale scaffold to address organism-specific regulation and nucleoid accessibility.

Beyond natural bacteria, this framework is well suited for synthetic-cell design. By prescribing compartment geometry and composition, one can input custom proteomes and gene sets and predict how engineered constituents reshape nucleoid organization and the accessibility of targeted chromosomal regions. This opens a route to rationally tuning genome compaction and expression capacity—for example, by engineering proteome charge balance to modulate nucleoid expansion, or by organizing translation components into DNA-depleted zones to create high-yield “expression factories.” While such applications will require system-specific parameterization, the underlying pipeline provides a versatile, extensible bridge from genotype to physiotype to phenotype.

Finally, our results emphasize how effective electrostatics, even when modeled implicitly, interact with crowding to shape genome-scale organization. Coupled with ion-explicit molecular dynamics, this framework can guide the design of synthetic or cell-free systems by informing choices of crowding agents and ionic conditions that better recapitulate *in vivo* behavior.

## Methods

### Intermolecular interactions

Proteins, ribosomes, and DNA beads are represented as interacting spheres suspended in a Newtonian cytosol, consistent with recent whole-cell coarse-grained models [8,70,99]. Nonbonded interactions comprise excluded-volume repulsion and (when enabled) screened electrostatics. Hard-sphere exclusion between particles *i* and *j* is

$$V_{ij}^{HS}(r_{i},r_{j})=\left\{ \begin{array}{cc} 0 & \text{if }r_{ij}>a_{i}+a_{j}\\ \infty & \text{if }r_{ij}\leq a_{i}+a_{j} \end{array}\right.,$$ (2)

where $r_{ij}=||r_{i}\;-\;r_{j}||$ is the distance between the two beads *i* and *j* centered at $r_{i}$ and $r_{j}$ with radii *a*_*i*_ and *a*_*j*_. A similar interaction potential is also used to model interactions between macromolecules and the cell membrane. In our *Brownian* simulations, $V_{ij}^{HS}$ is replaced by a steep, nearly hard repulsive Morse potential,

$$V_{ij}^{\text{morse}}(r_{i},r_{j})=\left\{ \begin{array}{cc} 0 & \text{if }r_{ij}>a_{i}+a_{j}\\ 6kT{\left(1-\exp\left(-60(r_{ij}-a_{i}-a_{j})/a_{\text{DNA}}\right)\right)}^{2} & \text{if }r_{ij}\leq a_{i}+a_{j} \end{array}\right.,$$ (3)

where *k* is Boltzmann’s constant and *T* is the absolute temperature. The potential in Eq (3) has been shown to recover hard-sphere behavior for volume fractions up to 50% [100,101].

We modeled the circular bacterial chromosome as a closed-loop bead-spring polymer chain, consistent with previous DNA modeling approaches [82,102]. The beads interact with each other via electrostatic attraction and repulsion, as well as hard-sphere exclusion, and also spring-like forces between base pairs and beads. The resistance to stretching and bending of the coarse-grained DNA chain is governed by spring and bending harmonic potentials, given, respectively, by

$$V_{ij}^{s}(r_{i},r_{j})=kT\frac{\sigma_{s}}{2\ell_{0}^{2}}(r_{ij}-\ell_{0}{)}^{2},$$ (4)

$$\;\text{and}\;V_{i}^{b}=kT\frac{\sigma_{b}}{2}(\theta_{i}-\theta_{i}^{0}{)}^{2},$$ (5)

where $\ell_{0}$ is the equilibrium bond length between a pair of adjacent DNA beads. In Eq (5), $\theta_{i}$ is the angle formed by a triplet of beads consisting of the DNA bead *i* and its neighbors, and $\theta_{i}^{0}$ is the triplet’s equilibrium angle (described in next section). The dimensionless coefficients $\sigma_{s}$ and $\sigma_{b}$ are the *stretching stiffness* and *bending stiffness*, respectively. For “bare” DNA (i.e., in the absence of NAPs binding to DNA), the equilibrium angle $\theta_{j}^{0}=\pi$ for all triplets throughout the entire chain. However, this equilibrium angle can be locally changed upon binding of bend-inducing HU proteins to the DNA [see Fig 5 and corresponding discussion].

Electrostatic interactions are modeled by a Debye–Hückel potential between particles with nominal charges *q*_*i*_ and *q*_*j*_ (see S1 Text for charge determination),

$$V_{ij}^{\text{elec}}=kT\frac{A_{ij}}{\kappa r}\exp\left(-\kappa (r_{ij}-a_{i}-a_{j})\right),$$ (6)

where *A*_*ij*_ is related to the net surface charges of particles *i* and *j* by [8,94]

$$A_{ij}=A_{i}A_{j},\text{where }A_{i}=\text{sign}\left(q_{i}\right)\sqrt{\frac{3}{4}q_{i,\text{eff}}},$$ (7)

where $q_{i,\text{eff}}=0.6\sqrt{\left|q_{i}\right|}\log(|q_{i}|/2\;+\;1)$ is the effective charge of the *i*-th particle and $\kappa^{-1}$ is the Debye length. This effective-charge mapping, introduced by Dutagaci et al. [94], provides a computationally efficient way to incorporate charge renormalization. Its sublinear dependence on |*q*_*i*_| mimics counterion-condensation (Manning-type) attenuation without explicitly simulating bound counterions.

Bare charges used to compute $q_{i,\text{eff}}$ are summarized in Table 1. Ribosome charge is adopted from Dutagaci et al. [94]. Cytoplasmic proteins are assigned class-average charges computed from the JCVI-Syn3A proteome (Fig A in S1 Text): $q_{\text{prot,+}}=+10\;e$ and $q_{\text{prot,-}}=-7\;e$. Each DNA bead represents a locally compacted 100-bp segment and is modeled as a sphere of radius 6 nm. Assigning the full phosphate charge of 100 bp (~−200e) to a 6 nm bead would unrealistically increase the local line-charge density. Instead, we assign each DNA bead the bare charge associated with a 12 nm backbone segment using DNA’s linear charge density (−5.9e/nm), yielding ~−70e per bead, and then apply the effective-charge mapping in Eq (7).

**Table 1:** Fixed parameters used in the Syn3A model. Values obtained through the pipeline in Fig A in S1 Text using data from Refs [79,93,94].

Quantity	Value	Notes	Source
Cell radius, *R*	225 nm	Standard; 201 nm in Fig 1	[70,79]
Stretching stiffness, $\sigma_{s}$	1	Dimensionless	[82]
Ribosome count	600	Standard; 503 in Fig 1	[70,79]
Protein radius, $a_{\text{prot}}$	3 nm		Fig A in S1 Text
Ribosome radius, $a_{\text{rib}}$	13 nm		[94]
DNA bead radius, $a_{\text{DNA}}$	6 nm	100 bp/bead	[82]
Debye length, $\kappa^{-1}$	0.68 nm	Fig 7; also 1.02 nm and 2.2 nm (Figs G and H in S1 Text)	[103]
DNA bead charge, $q_{\text{DNA}}$	−70e	Fig 7; also −200e and −400e (Figs G and H in S1 Text)	[93]
Protein charge, $q_{\text{prot,+}}$	+10e	Positively charged proteins	Fig A in S1 Text
Protein charge, $q_{\text{prot,-}}$	−7e	Negatively charged proteins	Fig A in S1 Text
Ribosome charge, $q_{\text{rib}}$	$-4\times 10^{3}\;e$		[94]

To assess sensitivity, we also examined two larger DNA bare-charge values in the S1 Text: (i) −200e, corresponding to assigning the full phosphate charge of 100 bp (straight-backbone assumption), and (ii) −400e, an upper-bound obtained by projecting an experimentally reported DNA surface charge density onto an equivalent total charge for a 6 nm spherical bead. These values are used to span electrostatic strengths rather than as equally physical representations.

Following Wennerström et al. [103], selective ion partitioning and macromolecular electroneutrality can increase the effective cytosolic Debye length; we therefore treat $\kappa^{-1}\approx 2.2$ nm as a plausible upper bound. As a lower bound, we compute $\kappa^{-1}$ for a representative monovalent ionic strength *I* = 0.2 M using the standard Debye–Hückel expression

$$\kappa^{-1}=\sqrt{\frac{\epsilon_{0}\epsilon_{r}kT}{2N_{A}Ie^{2}}},$$ (8)

we get $\kappa^{-1}\approx 0.68$ nm. Here, $\epsilon_{0}$ is the permittivity of free space, $\epsilon_{r}$ is the dielectric constant, *N*_*A*_ is the Avagadro number, and *e* is the electronic charge. Sensitivity of biomolecular organization across this range is examined in the S1 Text (Figs G and H in S1 Text). We have tabulated all the fixed parameters used in our JCVI-Syn3A model in Table 1.

### Swelling Monte Carlo algorithm

**Fig 8:**
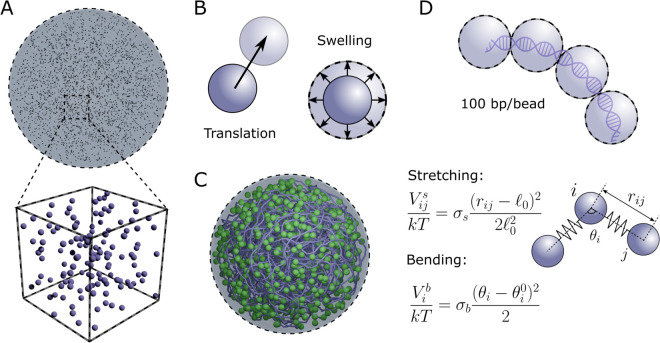
Illustration of the swelling Monte Carlo algorithm used to generate whole-cell configurations. (A) Macromolecules are initialized as point particles uniformly distributed throughout the cell and labeled by type (which determines their size and interaction potentials). (B) Particles then undergo two Monte Carlo moves: translation and swelling. (C) Resulting equilibrated whole-cell configuration. The chromosome is shown as a continuous backbone. (D) DNA beads (100 bp per bead, top) are connected through bead–spring and bending interactions (bottom), Eqs (4) and (5).

To generate the macromolecular distributions of ribosomes, proteins, and the chromosome in Syn3A, we use an extension of the swelling Monte Carlo algorithm developed in [97,104]. The method is illustrated in Fig 8. We initialize all macromolecules’ center positions in a uniform spatial distribution throughout the cell [Fig 8A]. We then apply alternating Monte Carlo translation and swelling moves to each particle to resolve overlaps [Fig 8B], to a physically consistent, dense configuration at the target packing fraction [Fig 8C]. The DNA is simultaneously self-assembled as it and the ribosomes take on their finite-size positions. Fig 8D illustrates the 100bp/bead coarse graining (top) and the stretching and bending potentials between DNA beads (bottom). Full algorithmic details, including parameter choices and convergence criteria, are provided in S1 Text.

Unlike approaches that prescribe macromolecular placement—for example, the Koch-curve chromosome construction used in [70]—this method does not require prior knowledge of experimentally measured macromolecular positions. Instead, macromolecular organization emerges from the specified physical interactions.

### Langevin dynamics

For our mesoscale dynamic simulations, the motion of the coarse-grained macromolecules in the cell is governed by Newton’s second law, resulting in a Langevin equation:

$$m\cdot \frac{dU}{dt}=F^{H}+F^{P}+F^{B},$$ (9)

where ***m*** is a diagonal mass matrix, U is the velocity of all particles, $F^{P}$ are the forces due to interparticle and particle-membrane interactions, given by

$$F_{i}^{P}=-\nabla_{r_{i}}V,$$ (10)

where

$$V=\sum_{i,j}(V_{ij}^{\text{morse}}+V_{ij}^{s}+V_{ij}^{\text{elec}})+\sum_{i}V_{i}^{b}$$ (11)

is the total potential energy of the system. Moreover, $F^{H}$ are the hydrodynamic forces, which, for spheres in the freely draining limit and in the absence of an external flow, are given by Stokes’ law $F^{H}=-6\pi \eta a\;\cdot \;U$, where a is a diagonal particle-radius matrix. Lastly, $F^{B}$ are the random forces and torques due to Brownian motion, given by $F^{B}=A\;\cdot \;\Psi$, where A is a positive-definite symmetric matrix and Ψ(t) is a white noise stochastic process, related to the hydrodynamic forces by the fluctuation-dissipation theorem.

⟨Ψ⟩=0, (12)

$$\langle \Psi (t_{0})\Psi (t_{0}+t)\rangle =\delta (t)I,$$ (13)

$$\text{and}A^{2}=2kT(6\pi \eta a),$$ (14)

where I is the identity matrix, and δ(t) is the Dirac delta distribution. Eq (9) is numerically integrated using the langevin integrator in LAMMPS for a Stokes number *St* = 10^−4^, chosen to approach the overdamped limit of the Langevin equation. Similar to [8], the timestep was chosen to be 42 ps. Interactions between the macromolecules and the cell membrane are modeled similar to [8] by adding an additional force to Eq (9) that act inward in the radial direction given by $F^{W}=-K(r_{i}-R-a_{i}{)}^{2}{\hat{e}}_{r}$ for $r_{i}=||r_{i}||\geq R-a_{i}$ and ${\hat{e}}_{r}=r_{i}/r_{i}$.

In our mesoscale simulations, we first equilibrated the initial distributions of DNA, ribosomes, and proteins in the absence of electrostatic interactions (Fig 6). We then enabled electrostatics, after which particles evolved under Brownian motion according to the Langevin dynamics in Eq (9), interacting via both excluded-volume (entropic) and charge-mediated forces.

### Radial distribution function for single and multiple realizations

To quantify the distribution of DNA and ribosomes throughout the cell, we introduce reduced probability densities P(r) for each particle type. Due to radial symmetry, the true ensemble distribution only depends on the radial coordinate r=||r|| and we write *P*(*r*). For a single numerical realization, the particle configuration is known and the probability density is given by

$$P(r)=\frac{1}{N}\sum_{k=1}^{N}\delta (r-r_{k}),$$ (15)

where *N* is the number of macromolecules of a given type and *δ* is the three-dimensional Dirac delta distribution. In this case, it is convenient to partition the cell domain into finite spherical shells of thickness *h* and calculate a shell-binned probability distribution, given by

$$P^{\text{sh}}(r)=\frac{1}{V_{\text{sh}}}\int_{V_{\text{sh}}}P(r)\;dV$$ (16)

This approach, similar to previous works in confined systems [7,105], quantifies the fraction of macromolecules whose centers are located in a spherical shell of radius *r* and thickness *h*, normalized by the shell volume. For our results, we use $h=2a_{\text{rib}}$. For a large number of particles N≫1 and small shell thickness *h*, this distribution approximates the real (ensemble) distribution function *P*(*r*).

For multiple runs, as we have many more available data points, the ensemble probability distribution *P*(*r*) can be better approximated by using spectral-decomposition methods [106]. To do so, we decompose *P*(*r*) in a series of orthonormal functions $\phi_{n}$ as $P(r)=\sum_{n}c_{n}\phi_{n}(r)$. The coefficients *c*_*n*_ can be calculated via orthogonal projection as:

$$c_{n}=\langle P,\phi_{n}\rangle =\int_{0}^{R}P(r)\phi_{n}(r)w(r)\;dr=\left\langle \frac{\phi_{n}(r)w(r)}{4\pi r^{2}}\right\rangle ,$$ (17)

where *w*(*r*) is the inner product weight and the angle brackets denote an ensemble average. For numerical purposes, we truncate the Fourier series at a finite number of basis functions. To calculate the coefficients *c*_*n*_, we use a Monte Carlo quadrature to approximate the ensemble average in Eq (17) by an arithmetic over multiple particles at multiple runs, as in the methodology proposed in [106]. More specifically, we use the even Chebyshev polynomials as basis functions when expanding *P*(*r*), for which $w(r)\propto 1/\sqrt{1-x^{2}}$.

### Calculation of average penetration length and traversability

The average penetration length $\langle L_{p}\rangle$ shown in Fig 4 is defined as

$$\langle L_{p}\rangle =\sum_{k}L_{p}(V_{k})P_{k},$$ (18)

where the summation is performed over all connected components of the Voronoi network for a particle of size *a*. *P*_*k*_(*a*) is the probability of a particle of such size being in the traversable region defined by the *k*-th connected component of the Voronoi network $V_{k}(a)$. As the Voronoi network provides a good representation of the accessible regions for a given particle of size *a*, we make a simplifying assumption that there is an equal likelihood of a particle to be in any of the accessible Voronoi edges, meaning that *P*_*k*_ is given by $N_{e}^{k}(a)/N_{e}(a)$, where $N_{e}^{k}(a)$ is the number of edges in $V_{k}(a)$ and *N*_*e*_(*a*) is the total number of Voronoi voids traversable by a particle of size *a*. Similarly, the traversability Tv(α), shown in Fig 4C, is defined as the probability of a particle being able to traverse a radial length *α* inside the cell. In Fig 4C, $\alpha =R_{95}$. Mathematically, this is given by

$$\text{Tv}(\alpha )=\mathbb{P}\{L_{p}(V_{k})\geq \alpha \}=\sum_{k}K(V_{k},\alpha )P_{k},$$ (19)

where the indicator function *K* is given by

$$K(V_{k},\alpha )=\left\{ \begin{array}{cc} 0 & \text{if }L_{p}(V_{k})<\alpha \\ 1 & \text{if }L_{p}(V_{k})\geq \alpha . \end{array}\right.$$ (20)

### Cluster size calculation

Cluster sizes in Fig H in S1 Text are computed using a graph-based (connected-components) algorithm, analogous to that used in our traversability analysis. We define an undirected contact network in which nodes represent beads and an edge connects particles *i* and *j* when their center-to-center separation satisfies $r_{ij}<c_{ij},c_{ij}=1.1\;(a_{i}+a_{j})$, where *a*_*i*_ and *a*_*j*_ are the bead radii.

## Supporting information

S1 Text Supplementary material and results. (PDF)
